# The association between zero-crossing temperatures and accidents due to icy conditions

**DOI:** 10.1177/14034948221148046

**Published:** 2023-04-04

**Authors:** Laura Maclachlan, Staffan Betnér, Tomas Lind, Antonis Georgelis, Mare Lõhmus

**Affiliations:** 1 Centre for Occupational and Environmental Medicine, Sweden; 2 Institute of Environmental Medicine, Karolinska Institutet, Sweden

**Keywords:** Climate change, temperature, falls, accidents, zero crossing, ice

## Abstract

**Aims:**

Rising temperatures lead to milder winters in Scandinavia. In certain regions, this could increase the number of winter days that fluctuate around 0°C (zero crossings). It has been frequently suggested that there is a higher risk of icy conditions during such days, which may lead to a predisposition to falls and road traffic accidents. Here, we examine the association between number of days with zero crossings and the number of hospitalisations and outpatient visits due to falls related to ice or snow or transport accidents.

**Methods:**

We used Poisson regression to examine the association between the number of days with zero crossings and the incidence of inpatient and outpatient visits related to falls due to ice and snow and to transport accidents during 2001–2017 in the Swedish cities of Stockholm, Malmö and Umeå.

**Results:**

We found a positive and significant association between the number of days of zero crossings and the number of in- and outpatient cases due to falls related to ice and snow. These associations were strongest in Umeå but less obvious in Stockholm and Malmö. In terms of injuries related to transport accidents, we saw a significant association between inpatient cases and number of zero crossings in Stockholm but not in Malmö or Umeå.

**Conclusions:**

**An increased number of zero crossings may increase out- and inpatient visits related to falls due to ice and snow or transport accidents. This effect is more pronounced in the northern city of Umeå than in Malmö, a city in Sweden’s southern-most region.**

## Introduction

Global warming is considered unequivocal [1]. Global surface temperatures increased by 0.85°C between 1880 and 2012 [2]. This is a trend observed during both summer and winter months, leading to heatwaves and milder winters. Temperatures in countries with temperate and cold climates, such as Sweden, have increased by up to 2°C over the last four decades [3]. One of the possible consequences of this in the northern parts of Sweden and the whole Northern European region is an increase in the number of days during winter when diurnal temperatures fluctuate around 0°C.

A ‘zero crossing’ is defined as a 24-hour period with a maximum temperature above 0°C and with a minimum temperature below 0°C [4]. The predictions conducted by Rossby Centre at the Swedish Meteorological and Hydrological Institute (SMHI) indicate that the number of zero crossings during winter in 2071–2100 will increase in the central and northern parts of Sweden compared to the period of 1971–2000 [4]. At the same time, the number of zero crossings in the southern part of the country is expected to decrease. The winters in northern Sweden today are characterised by sub-zero temperatures and a few days with zero crossings. As the temperature increases, the winter temperatures will approach zero, and more days with zero crossings may become possible. In southern Sweden, where the winter temperatures are already close to zero today, a warmer climate means fewer days with sub-zero temperatures and thus fewer days with zero crossings [4].

Temperature fluctuations around freezing point are associated with a higher risk of glaze or black ice, arising when precipitation that has fallen during plus temperatures freezes overnight [5]. This results in icy conditions that can predispose pedestrians to falls and lead to road traffic accidents. The incidence of fractures relating to falls has been shown to increase during the winter months [6,7], and nearly half of road traffic accidents in Sweden have been reported to occur during the three winter months [8].

Globally, injury relating to falls on ice or snow and transport accidents is a source of significant morbidity and mortality, causing 1.35 million deaths per year, constituting approximately 2% of all-cause mortality [9,10]. In Sweden, during the last two decades, about 3100 people per year have needed hospitalisation because of injuries caused specifically by falls due to ice and snow [11]. Many of these accidents are preventable. The associated economic costs are high. Road traffic accidents in Sweden have been estimated to cost €13.4 billion – approximately 2.6% of GDP [12]. Measures to prevent such events therefore have huge potential for a significant positive impact on public health.

### Aims

Various climate change–related risk assessments and reports, especially those conducted by different municipalities in Sweden [13,14], postulate that more days with zero crossings will increase morbidity related to accidents caused by ice or snow. However, as yet, there is no scientific evidence to support this theory. The aim of this study was to examine the association between the number of days where diurnal temperatures cross 0°C (zero crossings) and contact with outpatient and inpatient health care due to morbidity secondary to falls due to ice and snow and transport accidents.

## Methods

The study was conducted in the cities of Stockholm, Malmö and Umeå in Sweden (Supplemental Figure S1). These cities were selected to represent different geographic and climate regions. The study population comprised patients of all ages admitted to hospital between 2001 and 2017 as a result of morbidity secondary to falls due to ice and snow or a transport accident involving pedestrians, pedal cycles or motor vehicles.

Exposure data were obtained from the SMHI. Weather stations used were those with current and historic data in closest proximity to the study population and were Stockholm, Malmö and Umeå airport. Zero crossings were defined over 24-hour periods from 18:00 to 18:00, during which minimum and maximum air temperature included both minus and plus degrees Celsius, thereby crossing 0°C. Analysis was conducted for the winter months (October–April) when there is most diurnal variation across 0°C. Meteorological data used were minimum and maximum diurnal temperature, precipitation occurrence and amount and snow depth.

Outcome data were obtained from the Swedish National Board of Health and Welfare (Socialstyrelsen) using the National Patient Registries for outpatient and inpatient care (Patientregistret öppenvård and slutenvård) from 2001 to 2017. Codes for external causes of morbidity and mortality from the 10th revision of the International Classification of Diseases (ICD) were used. Cases of inpatient and outpatient care resulting from a fall due to ice and snow (W00) or transport accidents (V01–V79) were identified. Population data were obtained from Statistics Sweden (SCB).

The total numbers of days with a zero crossing within the time intervals of two, three, five and seven days were calculated. Truncated Poisson regression, performed in Stata v14.2 (StataCorp, College Station, TX), was used to analyse the association between the number of consecutive days with zero crossings within each time interval and the number of falls due to ice and snow (W00) or the number of transport accidents (V01–V79). Adjustment was made for precipitation occurrence, precipitation amount, snow depth, patient age and total population. We also present trend lines depicting the total number of days with zero crossings over time in Malmö, Stockholm and Umeå, based on the data available from the relevant weather stations (https://www.smhi.se/data/meteorologi/temperatur) in Supplemental Figures S2(a)–(c).

Ethics were sought via the Regional Ethical Review Board in Stockholm and were granted on 12 January 2017 (dnr 2016/2196-31). The study was conducted in accordance with the Declaration of Helsinki 2013 [15].

## Results

Demographic information for outpatient cases is reported in Table I and for inpatient cases in Table II. The study population was largest in Stockholm and smallest in Umeå, which is representative of the total number of residents in each city (Stockholm 952,058; Malmö 334,987; Umeå 125,434 [16]). The total number of falls due to ice and snow, leading to outpatient care, was 14,444 (Stockholm 9677; Malmö 1336; Umeå 3431), and the total leading to inpatient care was 5193 (Stockholm 3282; Malmö 576; Umeå 1335). The total number of cases of transport accidents leading to outpatient care was 44,630 (Stockholm 25,460; Malmö 13,485; Umeå 5685), and the total leading to inpatient care was 16,980 (Stockholm 9140; Malmö 5184; Umeå 2656). The age range of the total study population was 0–103 years. People seeking inpatient and outpatient care due to falls related to snow and ice had a higher median age than those seeking care for transport accidents.

**Table I.** Study population demographic information for outpatient cases.

	Stockholm		Malmö		Umeå	
	Fall accidents (N=9677)	Transport accidents (N=25,460)	Fall accidents (N=1336)	Transport accidents (N=13,485)	Fall accidents (N=3431)	Transport accidents (N=5685)
Age (years), range (median)	0–102 (52)	0–98 (36)	0–97 (50)	0–95 (35)	0–96 (53)	0–93 (31)
Age group, *n* (%)						
0–19	811 (8%)	3326 (13%)	224 (17%)	2481 (18%)	456 (13%)	1301 (23%)
20–39	2184 (23%)	10902 (43%)	285 (21%)	5270 (39%)	651 (19%)	2214 (39%)
40–59	3254 (34%)	8025 (32%)	377 (28%)	3607 (27%)	1017 (30%)	1393 (25%)
60–79	2775 (29%)	2722 (11%)	367 (27%)	1804 (13%)	1150 (34%)	674 (12%)
⩾80	653 (7%)	485 (2%)	83 (6%)	323 (2%)	157 (5%)	103 (2%)
Sex, *n* (%)						
Women	5692 (59%)	10321 (41%)	772 (58%)	6001 (45%)	2115 (62%)	2510 (44%)
Men	3985 (41%)	15139 (59%)	564 (42%)	7484 (55%)	1316 (38%)	3175 (56%)

**Table II.** Study population demographic information for inpatient cases.

	Stockholm		Malmö		Umeå	
	Fall accidents (N=3282)	Transport accidents (N=9140)	Fall accidents (N=576)	Transport accidents (N=5184)	Fall accidents (N=1335)	Transport accidents (N=2656)
Age, range (median)	1–102 (61)	0–99 (39)	1–97 (59)	0–103 (40)	1–99 (60)	0–97 (33)
Age group, *n* (%)						
0–19	200 (6%)	1544 (17%)	58 (10%)	1033 (20%)	159 (12%)	704 (27%)
20–39	417 (13%)	3125 (34%)	72 (13%)	1523 (29%)	169 (13%)	824 (31%)
40–59	937 (29%)	2558 (28%)	171 (30%)	1217 (23%)	336 (25%)	571 (21%)
60–79	1136 (35%)	1362 (15%)	180 (31%)	1011 (20%)	501 (38%)	429 (16%)
⩾80	592 (18%)	551 (6%)	95 (16%)	400 (8%)	170 (13%)	128 (5%)
**Sex, *n* (%)**						
Women	1859 (57%)	3407 (37%)	307 (53%)	2138 (41%)	748 (56%)	1048 (39%)
Men	1423 (43%)	5733 (63%)	269 (47%)	3046 (59%)	587 (44%)	1608 (61%)

Across all three cities, the 60–79 age group had the highest number of inpatient contacts for falls related to ice and snow, and the 0–19 age group had the lowest number of contacts. The number of outpatient contacts relating to falls was highest in the 40–59 age group in Stockholm and Malmö and in the 60–79 age group in Umeå, and it was lowest in the 0–19 age group in all three cities. The highest number of transport accidents was in the 20–39 age group across all three cities, and the lowest number was in the ⩾80 age group for both inpatient and outpatient contacts.

The association between the number days with zero crossings and the number of falls due to ice and snow is reported in Table III, which depicts this relationship during the time periods of two, three, five and seven consecutive days. The detected trends in associations were similar in both unadjusted (Supplemental Table S1(a)) and adjusted (Tables III and IV) models. A significant association between inpatient contacts due to falls related to ice and snow and the number of days with zero crossings was detected in Umeå when the data from two (or more) consecutive days were pooled. In Stockholm, this association appeared to be significant first when the data from five (or more) consecutive days were pooled (Table III; Supplemental Table S1(a)). There was a significantly increased risk of outpatient contacts due to falls related to ice and snow when data from two (or more) consecutive days were pooled in Umeå, and three (or more) consecutive days in Stockholm and Malmö.

**Table III.** Relationship between the number of falls related to ice and snow and the number of days with zero crossings within different time intervals (2 days, 3 days, 5 days and 7 days).

		**Inpatient cases**				**Outpatient cases**			
**Days**	Municipality	IRR	95% CI		p	IRR	95% CI		p
**2**	Malmö	1.23	0.71	2.11	0.460	1.16	0.85	1.60	0.351
	Stockholm	1.05	0.98	1.13	0.131	1.06	0.99	1.14	0.110
	Umeå	1.52	1.12	2.07	0.007*	1.25	1.07	1.47	0.006*
**3**	Malmö	1.11	0.85	1.46	0.447	1.21	1.00	1.46	0.045*
	Stockholm	1.05	0.99	1.11	0.084	1.08	1.01	1.15	0.018*
	Umeå	1.33	1.15	1.54	0.000*	1.23	1.07	1.42	0.004*
**5**	Malmö	1.02	0.80	1.30	0.884	1.25	1.06	1.48	0.009*
	Stockholm	1.09	1.05	1.14	0.000*	1.13	1.07	1.19	0.000*
	Umeå	1.25	1.12	1.41	0.000*	1.20	1.08	1.35	0.001*
**7**	Malmö	0.97	0.76	1.22	0.768	1.24	1.09	1.41	0.001*
	Stockholm	1.09	1.05	1.13	0.000*	1.09	1.04	1.15	0.001*
	Umeå	1.19	1.06	1.33	0.002*	1.17	1.08	1.28	0.000*

Models are adjusted for precipitation occurrence, precipitation amount, snow depth, patient age and total population. Significant results (95% CI) are marked with an asterisk.

IRR: incidence rate ratio; CI: confidence interval.

**Table IV.** Relationship between the number of transport accidents and the number of days with zero crossings within different time intervals (2 days, 3 days, 5 days and 7 days).

		**Inpatient cases**				**Outpatient cases**			
**Days**	Municipality	IRR	95% CI		p	IRR	95% CI		p
2	Malmö	1.01	0.84	1.23	0.884	0.96	0.89	1.04	0.324
	Stockholm	1.10	0.99	1.23	0.083	0.94	0.87	1.01	0.071
	Umeå	1.13	0.68	1.88	0.639	1.05	0.88	1.24	0.606
3	Malmö	0.96	0.85	1.09	0.572	1.03	0.96	1.10	0.409
	Stockholm	1.07	0.99	1.15	0.082	0.99	0.93	1.05	0.731
	Umeå	1.23	0.87	1.73	0.232	1.09	0.95	1.26	0.230
5	Malmö	1.03	0.93	1.13	0.607	1.03	0.98	1.09	0.256
	Stockholm	1.07	1.01	1.12	0.014*	1.03	0.98	1.09	0.246
	Umeå	0.92	0.73	1.16	0.494	1.08	0.98	1.20	0.120
7	Malmö	1.03	0.96	1.10	0.474	1.04	0.99	1.09	0.111
	Stockholm	1.08	1.04	1.12	0.000*	1.01	0.96	1.06	0.728
	Umeå	1.05	0.87	1.27	0.626	1.13	1.03	1.24	0.012*

Models are adjusted for precipitation occurrence, precipitation amount, snow depth, patient age and total population. Significant results (95 % CI) are marked with an asterisk.

IRR: incidence rate ratio; CI: confidence interval.

The association between transport accidents and the number of days with zero crossings is reported in Table IV. We detected a significant association between the number of zero crossings and inpatient contacts due to transport accidents in Stockholm when data from five or more consecutive days were pooled. No significant association was seen in Umeå and Malmö (Table IV; Supplemental Table SI(b)). There was a significant association between the number of outpatient contacts due to transport accidents and the number of days with zero crossings regarding data from seven consecutive days in Umeå. No significant associations were seen in Stockholm and Malmö.

## Discussion

We found a significant association between zero crossings and the number of falls related to ice and snow as well as between zero crossings and the number of injuries from road traffic accidents. These associations were more pronounced for falls relating to ice and snow than for injuries from road traffic accidents and for outpatient than for inpatient contacts. As pooling data over longer time intervals increases the statistical power, the trends observed became more obvious when longer time periods were analysed.

The associations between the number of inpatient cases resulting from falls related to ice and snow and the number of days with zero crossings were more easily detectable in the northern parts than in the southern parts of Sweden. Furthest north, in Umeå, the significant association between the number of days with zero crossings and the number of inpatient cases was detectable when data pooled over a period of two consecutive days were analysed. In Stockholm, the significant relationship between the number of inpatient cases and the number of days with zero crossings appeared first when data from five consecutive days were pooled. In Malmö, in the south of Sweden, no significant association between the number of days of zero crossing and inpatient contacts was detected.

A similar trend was also noticeable in the associations between outpatient visits due to falls related to ice and snow and the number of days with zero crossings. A significant association was apparent already when data from two consecutive days were pooled in Umeå, whereas in Stockholm and Malmö, a significant relationship between the exposure and outcome first appeared when data from three consecutive days were pooled. In general, the association between the number of falls related to ice and snow and the number of days with zero crossings was stronger for outpatient cases than for inpatient cases, which suggests that accidents resulting to injuries that do not require hospital care are more abundant than injuries that require hospital care.

The association between the number of inpatient cases related to road traffic accidents and days of zero crossings appeared to be significant when the associations were tested within five- and seven-day periods in Stockholm. This relationship was not significant in Malmö and Umeå and may be a reflection of generally heavier traffic in Stockholm. However, we did find a significant association between the number of outpatient contacts and the number of days with zero crossings when data from seven consecutive days were pooled in Umeå but not at the other study locations. It is possible that there is an association between the number of lighter traffic injuries and the occurrence of zero crossings in the northern parts of Sweden. However, as Umeå is a rather small city, the data may have been underpowered when the number of cases were pooled over shorter time intervals.

Our study results also showed that older age groups account for more outpatient and inpatient contacts due to falls on ice or snow. This is in keeping with falls in general, where older people have the highest risk of falls leading to serious injury or death [17]. A younger age group (20–39) had the highest number of outpatient and inpatient contacts due to transport accidents. This represents a global trend [18] and may reflect the fact that this group is more likely to be using forms of transport, such as cycling, in wintry conditions.

Supplemental Figures S2(a)–(c) show the time trend for total zero crossings per year in each of the study sites. If the trend, depicted in the figure, continues, we can expect that the number of days with zero crossings will decrease in Stockholm, remain largely constant in Malmö and increase in Umeå. These trend lines agree with the calculations conducted by Rossby Centre, which indicate that the number of zero crossings at the end of the century will increase in the northern parts of Sweden, where winters today are characterised by long periods with sub-zero temperatures, at the same time as it decreases or stays the same in the more southern parts of the country where winters are milder [4]. Thus, it is possible that climate change and rising temperatures will lead to a reduction in the number of zero crossings and related accidents in Stockholm, while in the more northern areas, an increase in zero crossings and possibly in the related accidents may occur in the future.

The strengths of this study include the fact that it explored the association between zero crossings and accidents for the first time. The study comprised a large cohort in three cities, allowing comparison between different geographic and climate areas. The study weaknesses include the fact that it was cross-sectional, and therefore causality cannot be determined. Furthermore, in this study, the same data were analysed multiple times pooled over different time intervals. This means that the presented results are not independent of each other. However, we chose to present data from all four different time intervals in order to illustrate the trends found in both unadjusted (Supplemental Table SI(a) and (b)) and adjusted models.

In 2006, the direct and indirect cost of falls to Swedish society exceeded US$2 billion – approximately 10% of Swedish health-care expenditure [19,20]. Falls related to ice and snow may account for up to 25% of these costs [21]. An increase in the number of days with zero crossings is likely to be associated with increased morbidity and economic burden in some parts of the country. A better understanding the risks associated with certain weather conditions will enable preventative planning in Sweden and in countries with similar climates to reduce both disease burden and economic costs.

## Supplemental Material

sj-docx-1-sjp-10.1177_14034948221148046 – Supplemental material for The association between zero-crossing temperatures and accidents due to icy conditions Supplemental material, sj-docx-1-sjp-10.1177_14034948221148046 for The association between zero-crossing temperatures and accidents due to icy conditions by Laura Maclachlan, Staffan Betnér, Tomas Lind, Antonis Georgelis and Mare Lõhmus in Scandinavian Journal of Public Health

sj-docx-2-sjp-10.1177_14034948221148046 – Supplemental material for The association between zero-crossing temperatures and accidents due to icy conditions Supplemental material, sj-docx-2-sjp-10.1177_14034948221148046 for The association between zero-crossing temperatures and accidents due to icy conditions by Laura Maclachlan, Staffan Betnér, Tomas Lind, Antonis Georgelis and Mare Lõhmus in Scandinavian Journal of Public Health

sj-docx-3-sjp-10.1177_14034948221148046 – Supplemental material for The association between zero-crossing temperatures and accidents due to icy conditions Supplemental material, sj-docx-3-sjp-10.1177_14034948221148046 for The association between zero-crossing temperatures and accidents due to icy conditions by Laura Maclachlan, Staffan Betnér, Tomas Lind, Antonis Georgelis and Mare Lõhmus in Scandinavian Journal of Public Health
