# Supplementary material for: The association between zero-crossing temperatures and accidents due to icy conditions
Source: Scand J Public Health. 2023 Apr 4;53(2):156–61. doi: 10.1177/14034948221148046 (PMC11907729; doi:10.1177/14034948221148046)
Supplement: sj-docx-1-sjp-10.1177_14034948221148046 – Supplemental material for The association between zero-crossing temperatures and accidents due to icy conditions [file sj-docx-1-sjp-10.1177_14034948221148046.docx]

**Title – Fig. 1S - Supplemental material for “The association between zero-crossing temperatures and hospital admissions due to falls on ice and snow and transport accidents.”**

**Description – Supplemental material, Fig. 1S, for “The association between zero-crossing temperatures and hospital admissions due to falls on ice and snow and transport accidents” by Maclachlan L, Lind T, Georgielis A, Lõhmus M in Scandinavian Journal of Public Health**


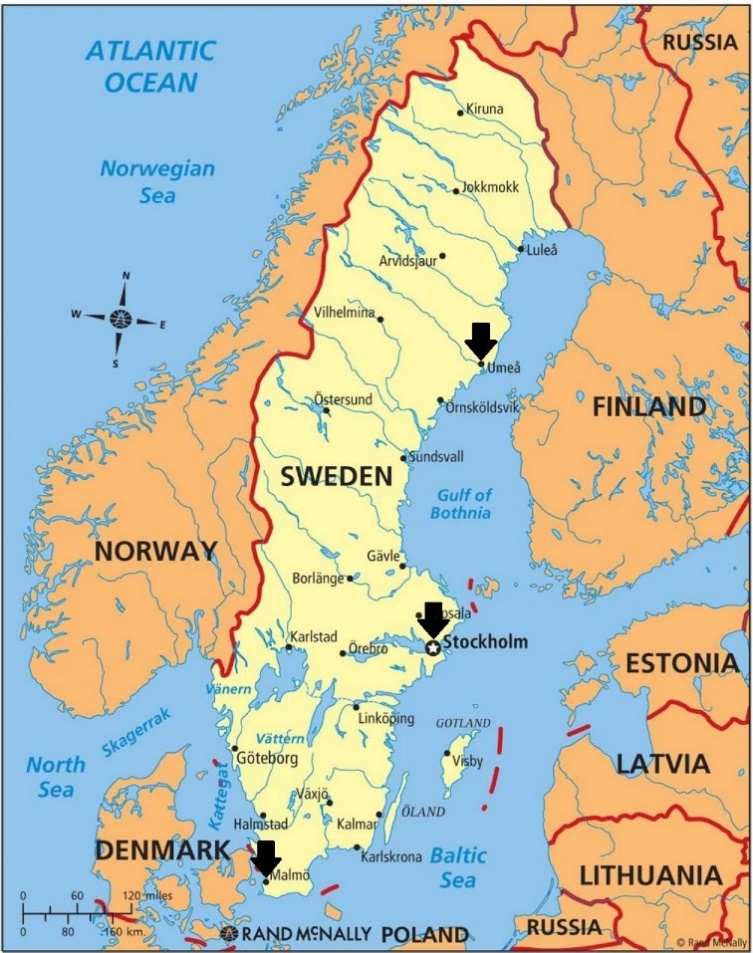
Fig. 1S Map showing the geographic position of Sweden in the nordic countries. Cities included in this study are marked with an arrow (Source: (maps-sweden.com)
