# Supplementary material for: The association between zero-crossing temperatures and accidents due to icy conditions
Source: Scand J Public Health. 2023 Apr 4;53(2):156–61. doi: 10.1177/14034948221148046 (PMC11907729; doi:10.1177/14034948221148046)
Supplement: sj-docx-2-sjp-10.1177_14034948221148046 – Supplemental material for The association between zero-crossing temperatures and accidents due to icy conditions [file sj-docx-2-sjp-10.1177_14034948221148046.docx]

**Title – Fig. 2S - Supplemental material for “The association between zero-crossing temperatures and hospital admissions due to falls on ice and snow and transport accidents.”**

**Description – Supplemental material, Fig. 2S, for “The association between zero-crossing temperatures and hospital admissions due to falls on ice and snow and transport accidents” by Maclachlan L, Lind T, Georgielis A, Lõhmus M in Scandinavian Journal of Public Health**

Fig. 2S Total zero crossings per year a) Stockholm from 1961 to 2018 with trend line b) Malmö from 1990 to 2018 with trend line c) Umeå from 1965 to 2018 with trend line


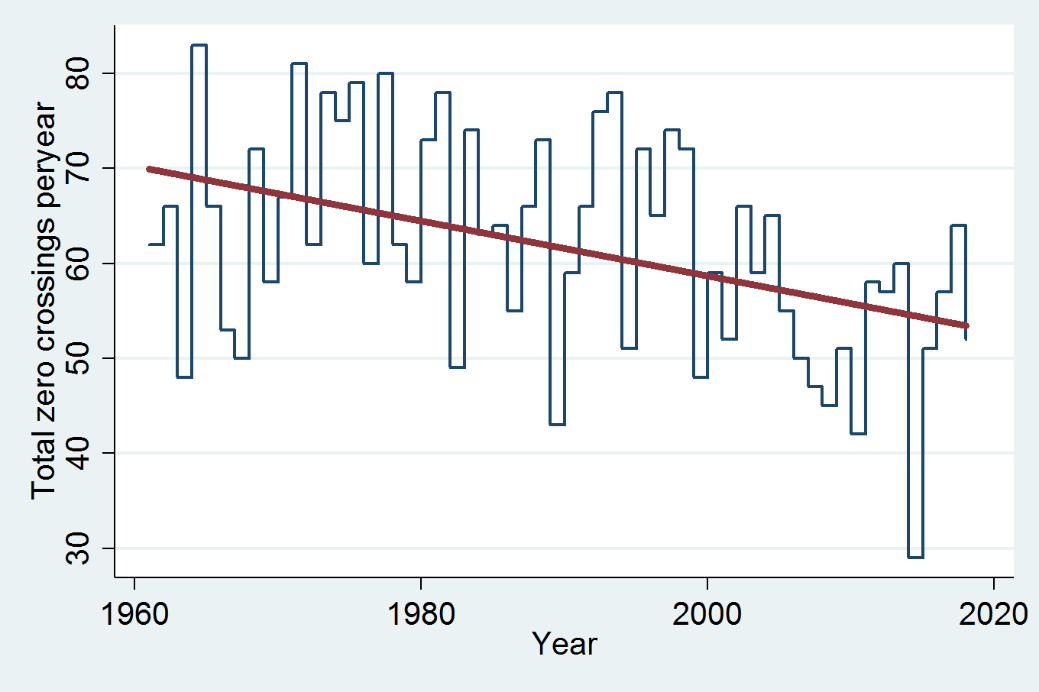
**a)**


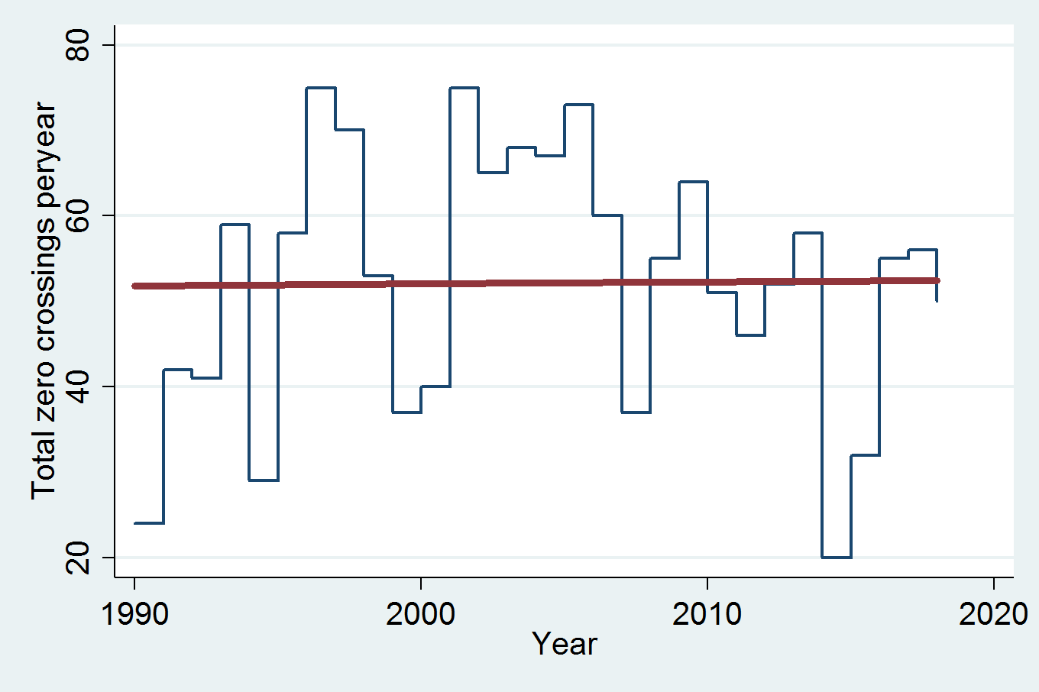
**b)**


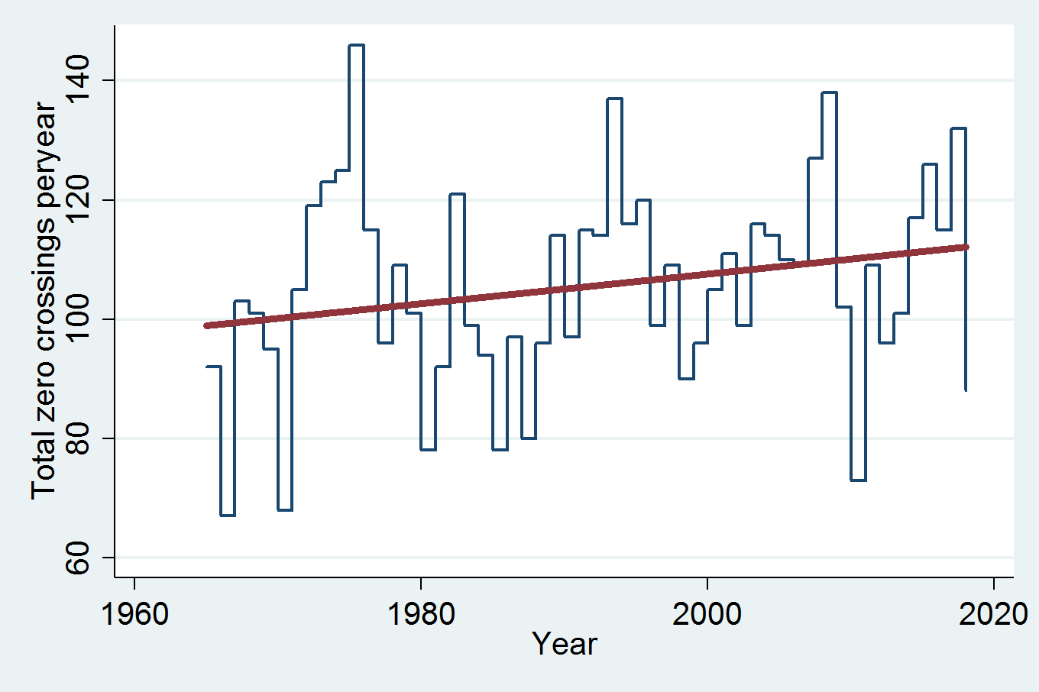
**c)**
