# Supplementary material for: The association between zero-crossing temperatures and accidents due to icy conditions
Source: Scand J Public Health. 2023 Apr 4;53(2):156–61. doi: 10.1177/14034948221148046 (PMC11907729; doi:10.1177/14034948221148046)
Supplement: sj-docx-3-sjp-10.1177_14034948221148046 – Supplemental material for The association between zero-crossing temperatures and accidents due to icy conditions [file sj-docx-3-sjp-10.1177_14034948221148046.docx]

## Supplementary materials

Table S1. A. Unadjusted relationship between the number of injuries related to ice and snow and the number of nights with zero crossings within different time intervals (2 days, 3 days, 5 days and 7 days). IRR – Incidence Rate Ratio; CI – Confidence Interval.

|  |  | Inpatient cases | | | | Outpatient cases | | | |
| --- | --- | --- | --- | --- | --- | --- | --- | --- | --- |
| days | Municipality | IRR | 95 % CI | | p < | IRR | 95 % CI | | p < |
| 2 | Malmö | 1.06 | 0.62 | 1.83 | 0.825 | 1.14 | 0.84 | 1.56 | 0.396 |
|  | Stockholm | 1.01 | 0.94 | 1.09 | 0.699 | 1.02 | 0.95 | 1.11 | 0.546 |
|  | Umeå | 1.52 | 1.12 | 2.06 | 0.007 | 1.23 | 1.04 | 1.46 | 0.015 |
| 3 | Malmö | 1.08 | 0.84 | 1.39 | 0.558 | 1.17 | 0.97 | 1.40 | 0.100 |
|  | Stockholm | 1.03 | 0.96 | 1.09 | 0.519 | 1.06 | 0.98 | 1.15 | 0.117 |
|  | Umeå | 1.37 | 1.17 | 1.60 | 0.000 | 1.25 | 1.09 | 1.44 | 0.001 |
| 5 | Malmö | 1.01 | 0.80 | 1.29 | 0.913 | 1.20 | 1.02 | 1.42 | 0.027 |
|  | Stockholm | 1.07 | 1.02 | 1.13 | 0.009 | 1.13 | 1.06 | 1.20 | 0.000 |
|  | Umeå | 1.29 | 1.16 | 1.43 | 0.000 | 1.26 | 1.14 | 1.40 | 0.000 |
| 7 | Malmö | 0.97 | 0.78 | 1.20 | 0.778 | 1.22 | 1.08 | 1.39 | 0.002 |
|  | Stockholm | 1.09 | 1.04 | 1.15 | 0.000 | 1.11 | 1.34 | 1.19 | 0.003 |
|  | Umeå | 1.19 | 1.08 | 1.32 | 0.001 | 1.21 | 1.10 | 1.32 | 0.000 |

Table S1. B. Unadjusted relationship between the number of injuries related traffic accidents and the number of nights with zero crossings within different time intervals (2 days, 3 days, 5 days and 7 days). IRR – Incidence Rate Ratio; CI – Confidence Interval

|  |  | Inpatient cases | | | | Outpatient cases | | | |
| --- | --- | --- | --- | --- | --- | --- | --- | --- | --- |
| days | Municipality | IRR | 95 % CI | | p < | IRR | 95 % CI | | p < |
| 2 | Malmö | 1.03 | 0.85 | 1.25 | 0.765 | 0.94 | 0.87 | 1.02 | 0.140 |
|  | Stockholm | 1.11 | 0.99 | 1.24 | 0.069 | 0.94 | 0.87 | 1.01 | 0.082 |
|  | Umeå | 1.10 | 0.66 | 1.84 | 0.713 | 1.05 | 0.88 | 1.26 | 0.591 |
| 3 | Malmö | 0.98 | 0.87 | 1.10 | 0.698 | 1.00 | 0.93 | 1.07 | 0.921 |
|  | Stockholm | 1.06 | 0.99 | 1.5 | 0.105 | 0.98 | 0.21 | 1.04 | 0.533 |
|  | Umeå | 1.16 | 0.86 | 1.57 | 0.331 | 1.11 | 0.95 | 1.29 | 0.178 |
| 5 | Malmö | 1.02 | 0.93 | 1.12 | 0.680 | 1.00 | 0.95 | 1.06 | 0.921 |
|  | Stockholm | 1.06 | 1.06 | 1.11 | 0.027 | 1.02 | 0.97 | 1.08 | 0.471 |
|  | Umeå | 0.89 | 0.71 | 1.12 | 0.314 | 1.15 | 1.02 | 1.28 | 0.018 |
| 7 | Malmö | 1.02 | 0.95 | 1.09 | 0.672 | 1.03 | 0.98 | 1.08 | 0.311 |
|  | Stockholm | 1.08 | 1.04 | 1.13 | 0.000 | 1.02 | 0.97 | 1.08 | 0.452 |
|  | Umeå | 1.07 | 0.85 | 1.22 | 0.853 | 1.17 | 1.05 | 1.30 | 0.005 |
